# Does business news sentiment matter in the energy stock market? Adopting sentiment analysis for short-term stock market prediction in the energy industry

**DOI:** 10.3389/frai.2025.1559900

**Published:** 2025-07-23

**Authors:** Chi-Yuan Lee, Eva Anderl

**Affiliations:** HM Business School, Hochschule München University of Applied Sciences, Munich, Germany

**Keywords:** energy industry, stock prediction, sentiment analysis, news sentiment, LSTM, FinBERT

## Abstract

Characterized by high volatility the energy stock market provides ample research potential for stock market prediction using machine learning models. This paper investigates using business news as an indicator of market sentiment in Recurrent Neural Networks. The authors adopt a finance-specific Transformer-based model, FinBERT, for news sentiment analysis and use a Long Short-Term Memory (LSTM) model for stock prediction. As prior research indicates that sentiment may vary for different news elements, they specifically explore differences between news headlines and content. Results show that (1) transformer-based sentiment analysis of business news can improve stock market prediction in the energy industry and that (2) sentiment of news content is more effective than sentiment of news headlines.

## 1 Introduction

Predicting stock market prices is a well-known challenge in machine learning due to the complex and dynamic nature of financial markets (Rouf et al., 2021). Various data sources have been explored to predict market performance, including technical factors and macroeconomic indicators (Latif et al., 2025) as well as global crises (Nuta et al., 2024). Over the last years, market sentiment, i.e., investors’ emotions toward a specific target affecting their decision-making (Medhat et al., 2014), has gained attention as a valuable predictor. Social media (Herrera et al., 2022; Reboredo and Ugolini, 2018) and news (Gupta and Banerjee, 2019; Li et al., 2021) have been identified as relevant sources influencing investors’ emotions—especially as interaction frequency with online content increases.

Sentiment analysis, a subfield of natural language processing (NLP), allows to computationally determine the emotional tone of digital textual content (Liagkouras and Metaxiotis, 2024), thereby supporting its integration into stock market prediction models. As of late, Transformer models have emerged as a promising advancement in NLP (Rahali and Akhloufi, 2023). Transformer models are neural networks that leverage the self-attention mechanism and have demonstrated superior performance over traditional models across various natural language processing tasks, including speech recognition and machine translation (Rahali and Akhloufi, 2023). In sentiment analysis, they have demonstrated superior performance in capturing contextual meaning and complex linguistic patterns (Mishev et al., 2020).

Prior research shows that market sentiment features derived using sentiment analysis can enhance model performance for stock prediction in different industries (Jin et al., 2020; Sarkar et al., 2020) and for market indexes (Li et al., 2014; Shi et al., 2018). Yet, as machine learning approaches for stock prediction are not always directly applicable to other industries (Ebadi et al., 2019), this study aims to find out whether sentiment analysis with novel Transformer-based models can be used for short-term stock market prediction in a highly volatile industry such as the energy sector. Characterized by its high volatility, the energy sector of the stock market is an interesting field of application for predicting stock market movements. According to Moran (2020), the energy sector exhibits the highest volatility compared to the commodities, financial, and technology sector from 2009 to 2019. This volatility is also reflected in a highly volatile annual return. For instance, the energy sector in S&P 500 fluctuated −33.68%, 54.64%, 65.72% and −1.33% in 2020, 2021, 2022 and 2023 respectively (US Bank, 2024).

Selected studies have already examined the use of social media sentiment for energy stock prediction (Ben Yahia et al., 2024; Herrera et al., 2022; Reboredo and Ugolini, 2018). However, there is a lack of research on sentiment analysis of general business news in the energy industry. Studies in other domains have shown that business news can have an impact on stock prediction (Deveikyte et al., 2022; Li et al., 2014; Ranco et al., 2016; Sarkar et al., 2020). For example, Deveikyte et al. (2022) find evidence of correlation between sentiment in tweets and news headlines and stock market movements for the largest 100 companies listed on the London Stock Exchange using VADER, a lexicon and rule-based sentiment analysis tool. Yet, to the best of our knowledge, the application of Transformer-based models for sentiment analysis of general business news in the context of industry-specific stock market prediction remains unexplored.

Given that news headlines and news content serve different purposes for both publishers and readers, we propose to analyze them separately as their sentiment and predictive capability may differ. Especially in a digital environment, news headlines are an important tool to attract the readers’ attention. They are supposed to raise the curiosity of readers to entice them to open the article. Prior research has shown that containing sentimental words significantly increases the click-through performance of headlines (Kuiken et al., 2017), which might prompt editors to formulate headlines with different sentiment.

Taken together, this paper will analyze the following research questions to investigate the applicability of Transformer-based sentiment analysis of business news in the energy industry:

1. Can Transformer-based sentiment analysis of business news improve short-term stock market prediction in the energy industry?
2. Do news headlines and content differ in their predictive capabilities?

## 2 Methods

To address our research questions, we conduct a quantitative study using real-world data. Our dataset comprises approximately 10 years of data from 7 energy sector companies. We employ FinBERT, a finance-specific Transformer-based model, for news sentiment analysis and utilize a Long Short-Term Memory (LSTM) model for stock price prediction.

### 2.1 Data collection

For our research, we select 7 energy companies listed on the New York Stock Exchange (NYSE) with top-ranking capital values according to the S&P Energy Index in November 2022: Exxon Mobil (XOM), Chevron Corporation (CVX), ConocoPhillips (COP), Eog Resources (EOG), Schlumberger N. V. (SLB), Occidental Petroleum Corp (OXY), and Pioneer Natural Resources Company (PXD). The research time interval is set from January 2013 to November 2022, covering 9 years and 11 months in total. This time interval is considerably longer than the average time period covered in prior stock market prediction studies (Kumar et al., 2021), allowing for a comprehensive analysis.

Stock data and business news are collected separately. Stock data are collected via the Yahoo Finance open source API (GitHub, 2023) with all features in numerical form. The stock data contains five basic features from historical trades, namely open, high, low, close, adjusted close, and volume. Open refers to the open price of the trading day, high refers to the highest price within the trading day, low refers to the lowest price of the trading day, close refers to the close price of the trading day, adjusted close refers to the close price with the consideration of dividend payoff on a certain date, and volume refers to the number of trades of the trading day.

The collection of textual data is more complex. We design a web scraping program to collect news pages within the research time interval automatically. The program extracts the publication timestamps, publishers, news headlines, and news content from 10 selected news publishers with widespread readership covering global financial markets and corporate affairs (Bloomberg, CNBC, The Economist, Financial Times, Forbes, The New York Times, Reuters, The Wall Street Journal, The Washington Post, and Yahoo Finance). In total, we collect 18,254 news headlines and corresponding URLs from Google News (2025) using Python. Utilizing the requests library, we then retrieve the full HTML content for 17,862 of these articles, resulting in a dataset comprising 17,862 complete data points.

### 2.2 Data preprocessing

In the data preprocessing stage, stock data and business news are subjected to different processing approaches. For stock data, we select close, open, and volume as the basic information in each stock. We normalize each close, open, and volume between 0 and 1 for each stock, then, we aggregate the 7 normalized stocks as an index’s close, index’s open, and index’s volume. The purpose of creating an index is to simplify and overcome lack of data from individual company stocks. Then, we create three extra features, percent, diff, and fluctuation to represent the differences of index close and index open.

For business news, we first remove duplicate entries based on identical URLs, followed by the elimination of HTML codes and irrelevant information such as advertisements, suggested readings, article information, and unrelated context. To identify passages relevant to our selected companies, we tokenize each article into individual sentences. For each sentence containing the target keyword, we extract the surrounding five sentences. After removing overlapping segments to avoid duplication, the resulting snippets are used as input for sentiment analysis.

To perform the sentiment analysis, we use FinBERT (Huang et al., 2023), a specialized financial language model based on BERT (Bidirectional Encoder Representations from Transformers), designed for natural language processing tasks in finance. FinBERT has been trained on financial texts, such as analyst reports, financial news, and SEC filings, to enhance its understanding of financial terminology and sentiment. It has been shown to substantially outperform other machine learning algorithms in sentiment classification (Huang et al., 2023). Prior researchers have already successfully used FinBERT in sentiment analysis of summarized news extracted from *The New York Times* (Kim et al., 2023).

Using the five-sentence snippets as input, the final layer of FinBERT produces a softmax output, yielding a probability distribution across three sentiment classes: positive, negative, and neutral. The sum of the three categories equals 1.^1^ Using this classification, we create six aggregate sentiment features for each day: headline_positive, headline_negative, headline_neutral, content_positive, content_negative, and content_neutral.

As 1,373 samples have no news sentiment collected on that date, our final training dataset consists of 2,497 samples with 11 features and 1 target variable (i.e., index_close). Table 1 provides an overview of the features in our final dataset.

**Table 1:** Descriptive statistics of features.

Summary statistics	Index_open	Index_close	Volume	Percentage	Diff	Headline_positive	Headline_neutral	Headline_negative	Content_positive	Content_neutral	Content_negative
Count	2,497	2,497	2,497	2,497	2,497	1,124	1,124	1,124	1,124	1,124	1,124
Mean	3.350	3.340	0.821	0.003	−0.010	0.516	1.762	0.547	0.936	1.182	0.706
SD	1.100	1.104	0.462	0.102	0.066	0.920	1.786	0.798	1.504	1.242	0.912
Min	0.082	0.044	0.026	−0.840	−0.467	0.007	0.015	0.007	0.006	0.012	0.007
25%	2.811	2.802	0.514	−0.013	−0.048	0.060	0.825	0.033	0.065	0.300	0.035
Median	3.485	3.476	0.694	0.001	−0.011	0.166	0.942	0.177	0.414	0.908	0.373
75%	4.072	4.059	1.003	0.014	0.025	0.681	2.186	0.861	1.070	1.522	0.976
Max	5.947	5.999	5.022	3.416	0.356	11.583	18.066	5.480	20.330	9.986	6.465

The count of samples excludes missing values. *n* = 100.

As shown in Table 1, neutral sentiment dominates in both headlines and content. In headlines, negative sentiment exceeds positive sentiment, whereas in content, positive sentiment is more prevalent than negative. Besides, we observe that the distributions of sentiment features are all left-skewed, whereas stock price features are more symmetric.

After preparing the dataset, we create five subsets of features for performance comparison between models with and without sentiment features (see Table 2). Our baseline is the subset only including the stock price features. Subset 1 contains stock price features and content sentiment features. Subset 2 contains stock price features and headline sentiment features. Subset 3 includes stock price features, content sentiment features, and headline sentiment features. Subset 4 contains only headline and content sentiment features.

**Table 2:** Subset details.

Features	Baseline	Subset 1	Subset 2	Subset 3	Subset 4
Index_open	X	X	X	X	
Volume	X	X	X	X	
Percent	X	X	X	X	
Diff	X	X	X	X	
Fluctuation	X	X	X	X	
Headline_positive			X	X	X
Headline_neutral			X	X	X
Headline_negative			X	X	X
Content_positive		X		X	X
Content_neutral		X		X	X
Content_negative		X		X	X

### 2.3 Model architecture and training

A popular approach in stock-market prediction is using Recurrent Neural Networks (RNNs), which have become state-of-the-art models for a variety of machine learning problems (Greff et al., 2017). Specifically, RNNs with Long Short-Term Memory are a frequently used tool in time series forecasting (Guo, 2020; Jin et al., 2020; Sarkar et al., 2020). As LSTMs have been successfully used for stock prediction with market sentiment (Bhandari et al., 2022; Fischer and Krauss, 2018; Sarkar et al., 2020; Yadav et al., 2020), we adapt this approach and use an LSTM model.

We employ a five-layer LSTM architecture, each with 50 hidden units, followed by a dropout layer with a dropout rate of 0.2 after every LSTM layer. A dense layer with 1 hidden unit is added at the end of the model. The hyperparameters were determined during training using a grid search.

For model training, we split the whole dataset into training, validation, and test datasets (70, 15, and 15%). Since we are dealing with time-series data, we do not shuffle the samples so that the sequence of the samples remains. All models are trained with 60 timestamps and predict the 61^st^ target variable. To prevent overfitting, we use a checkpoint to reserve the best epoch in iterations based on the lowest validation loss. Finally, we use the test data as out of sample dataset to evaluate the final results. Besides, because of the stochastic nature of LSTMs, the model performance might vary even with the same hyperparameters. To examine and compare the true performances, we retrain and evaluate each model 100 times to collect the performance distributions. As a robustness check, we add an alternative implementation using XGBoost (Chen and Guestrin, 2016), a widely used machine learning algorithm.

## 3 Results

For model evaluation, we use Mean Absolute Error (MAE), Mean Absolute Percentage Error (MAPE), Mean Square Error (MSE) and Root Mean Squared Error (RMSE). A review of existing research indicates that MSE is most frequently employed for stock market prediction, closely followed by Accuracy and MAE (Ketsetsis et al., 2020). In another systematic literature review, it was found that RMSE, MAPE, and MSE were the most frequently employed metrics (Nti et al., 2020).

As we retrain each model 100 times, we obtain the distribution in each subset. Besides, we also apply statistical tests calculating the z-score and *p*-value for MAE to examine if there are significant differences when using the sentiment features.

As shown in Table 3, for the LSTM, average performances in subset 1 (stock price features + news content) are better than in the baseline relying only on stock price features. The model performs better in subset 3 (stock price features + news content + news headlines) than in subset 2 (stock price features + news headlines), whereas in subset 4 (news content + news headlines), which includes only sentiment scores and no stock price features, the model does not perform well.

LSTM and XGBoost achieve similar predictive accuracy (MAE, MAPE, MSE, and RMSE). Yet, interestingly, XGBoost seems to rely less on sentiment features, with the baseline model outperforming the other models. However, the ranking of sentiment-based models (subset 1 performing better than subset 2 and 3) remains consistent with the LSTM results across all metrics, supporting the robustness of our results.

**Table 3:** Model performance distributions.

Subsets	MAE			MAPE			MSE			RMSE		
	LSTM		XGBoost	LSTM		XGBoost	LSTM		XGBoost	LSTM		XGBoost
	Mean	SD		Mean	SD		Mean	SD		Mean	SD	
Baseline	0.184	0.038	0.166	0.092	0.058	0.054	0.053	0.022	0.051	0.230	0.024	0.226
Subset 1	0.168	0.028	0.170	0.084	0.030	0.055	0.047	0.015	0.053	0.217	0.017	0.230
Subset 2	0.176	0.087	0.175	0.084	0.037	0.057	0.059	0.131	0.055	0.243	0.135	0.235
Subset 3	0.175	0.026	0.174	0.085	0.034	0.056	0.051	0.017	0.055	0.226	0.019	0.235
Subset 4	1.665	0.154	1.217	0.437	0.123	0.335	3.704	0.645	1.913	1.925	0.084	1.383

To compare performance differences among the subsets, we use statistical tests to verify if the sentiment features bring a significant improvement in average LSTM performance. We compare the mean model performance in the baseline and the mean model performance for other subsets using MAE, whose scale depends on the scale of the data, thus making it easy to interpret if applied to a single dataset (Hyndman and Koehler, 2006). Table 4 shows the statistical test results for the LSTM models.^2^

**Table 4:** Statistical test results (LSTM).

Subsets	Compare to baseline	
	Z-score	*P*-value
Baseline	0.000	0.500
Subset 1	−3.286	0.001^***^
Subset 2	−0.775	0.219
Subset 3	−1.775	0.038^*^
Subset 4	92.972	1.000

*p ≤ 0.05; **p ≤ 0.01; ***p ≤ 0.001. *n* = 100.

In light of Table 4, compared to the baseline, the performance improvements in subset 1 (*p* = 0.001) and subset 3 (*p* = 0.038) are statistically significant. Thus, we conclude that for the LSTM, subset 1 (stock price features and news content) and subset 3 (stock price features, news content, and news headlines) significantly outperform the baseline.

## 4 Discussion

In this paper, we examine whether business news sentiment features created with FinBERT can be used in Transformer-based stock price prediction in the energy industry. Combining stock price features with news sentiment features significantly improves the average predictive performance for LSTM models. Adopting subset 1 (stock price features and content sentiment features) is the most effective combination. In comparison, headline sentiment features seem to be less effective.

Our findings have several important theoretical implications. First, we show that sentiment analysis of business news can successfully be applied in the energy sector. While social media sentiment has been used for energy stock prediction (Ben Yahia et al., 2024; Herrera et al., 2022; Reboredo and Ugolini, 2018), general business news have not been tested in the energy sector. Second, we provide an example of how domain-specific Transformer models can be applied in sentiment analysis. While FinBERT has already been shown to outperform more established analysis approaches regarding classification accuracy of central bank communication (Kim et al., 2024), applications in an industry-specific context are still rare. As the data preparation steps taken are not specific to the energy industry, our paper provides a blueprint of how to apply sentiment analysis of general business news for stock market prediction in different industry settings. Third, we show that news content and news headlines differ in their predictive ability. A potential explanation is that news headlines might include some overreacted sentiment to attract the readers’ eyes (Rieis et al., 2021). Prior studies support the existence of the incongruity between headlines and content (Deveikyte et al., 2022; Yoon et al., 2019; Yoon et al., 2021). Yet, unlike Deveikyte et al. (2022), our study finds that for LSTMs, news content is more effective than news headlines. This divergence might be explained by three reasons: (1) in contrast to Deveikyte et al. (2022), our study uses the same data source for headlines and news, thus eliminating the risk of potential differences in the datasets. (2) We analyze general business news and not financial news and focus on the energy industry. Writing styles and thus sentiment distribution might differ between these news types and within industries. (3) The importance of features may differ between traditional machine learning models (such as XGBoost) and deep learning models (Lai et al., 2019). Collectively, our findings thus highlight the importance of analyzing headlines and content independently and show a need for more differentiated research. Content features, requiring more effort to extract and thus less commonly utilized in existing research, provide additional value and should not be neglected.

From a practical perspective, our study has implications for several groups interested in stock market prediction. While adopting sentiment features as the only feature set is not sufficient to predict stock prices (as in subset 4), Transformer-based sentiment analysis of business news can help to improve stock prediction performance in the energy industry, especially when analyzing headlines and content separately. Quantitative traders can thus introduce business news sentiment features generated by domain-specific Transformer models in trading models. Additionally, this research also points out a risk of stock market manipulation using news sentiment. Regulatory authorities should monitor this closely and might need to update policies and regulation accordingly.

As every research, our study is subject to several limitations. First, we collected data from 10 news publishers and 7 energy companies from January 2013 to November 2022. Even though this provides us with sufficient data to conduct our analyses, we suggest future studies expand the scope of data sources and extend the time interval. Second, our research could be expanded by testing other types of stock prediction such as percentage of return, stock picking, and, potentially, a swing trade strategy. Third, we focused on one LSTM specification and FinBERT as a single sentiment analysis model. We suggest further researchers adopt other models and apply different sentiment analysis approaches as outlined by Liagkouras and Metaxiotis (2024) to investigate the strengths and weaknesses of different approaches.

## Data Availability

The raw data supporting the conclusions of this article will be made available by the authors, without undue reservation.

## Footnotes

^1^For details about the implementation of FinBERT, please refer to Liu et al. (2021).

^2^For XGBoost, a significance test is not possible.

## References

[ref1] Ben Yahia S. Garcia Sanchez J. A. Kaffel R. H. (2024). Impact of sentiment analysis on energy sector stock prices: a FinBERT approach [Preprint].

[ref2] Bhandari H. N. Rimal B. Pokhrel N. R. Rimal R. Dahal K. R. Khatri R. K. (2022). Predicting stock market index using LSTM. Mach. Learn. Appl. 9:100320. doi: 10.1016/j.mlwa.2022.100320

[ref3] Chen T. Guestrin C. (2016). “XGBoost: A scalable tree boosting system,” in Proceedings of the 22nd ACM SIGKDD International conference on knowledge discovery and data mining. New York, NY: Association for Computing Machinery, 785–794. doi: 10.1145/2939672.2939785

[ref4] Deveikyte J. Geman H. Piccari C. Provetti A. (2022). A sentiment analysis approach to the prediction of market volatility. Frontiers in Artificial Intelligence 5:836809. doi: 10.3389/frai.2022.836809, PMID: 36620753 PMC9815756

[ref5] Ebadi A. Gauthier Y. Tremblay S. Paul P. (2019). “How can automated machine learning help business data science teams?” in *2019 18th IEEE International Conference On Machine Learning And Applications (ICMLA)*, 1186–1191.

[ref6] Fischer T. Krauss C. (2018). Deep learning with long short-term memory networks for financial market predictions. Eur. J. Oper. Res. 270, 654–669. doi: 10.1016/j.ejor.2017.11.054

[ref7] GitHub. (2023). Ranaroussi/yfinance: download market data from yahoo! Finance's API. Available online at: https://github.com/ranaroussi/yfinance (accessed September 14, 2023).

[ref8] Google News. (2025). Google news. Available online at: https://news.google.com/home?hl=de&gl=DE&ceid=DE:de (accessed January 8, 2025).

[ref9] Greff K. Srivastava R. K. Koutnik J. Steunebrink B. R. Schmidhuber J. (2017). LSTM: a search space odyssey. IEEE Trans. Neural Netw. Learn. Syst. 28, 2222–2232. doi: 10.1109/TNNLS.2016.2582924, PMID: 27411231

[ref10] Guo Y. (2020). “Stock price prediction based on LSTM neural network: the effectiveness of news sentiment analysis,” in *2020 2nd International Conference on Economic Management and Model Engineering (ICEMME)*, 1018–1024.

[ref11] Gupta K. Banerjee R. (2019). Does OPEC news sentiment influence stock returns of energy firms in the United States? Energy Econ. 77, 34–45. doi: 10.1016/j.eneco.2018.03.017

[ref12] Herrera G. P. Constantino M. Su J.-J. Naranpanawa A. (2022). Renewable energy stocks forecast using twitter investor sentiment and deep learning. Energy Econ. 114:106285. doi: 10.1016/j.eneco.2022.106285

[ref13] Huang A. H. Wang H. Yang Y. (2023). FinBERT: a large language model for extracting information from financial text. Contemp. Account. Res. 40, 806–841. doi: 10.1111/1911-3846.12832

[ref14] Hyndman R. J. Koehler A. B. (2006). Another look at measures of forecast accuracy. Int. J. Forecast. 22, 679–688. doi: 10.1016/j.ijforecast.2006.03.001

[ref15] Jin Z. Yang Y. Liu Y. (2020). Stock closing price prediction based on sentiment analysis and LSTM. Neural Comput. & Applic. 32, 9713–9729. doi: 10.1007/s00521-019-04504-2

[ref16] Ketsetsis A. P. Kourounis C. Spanos G. Giannoutakis K. M. Pavlidis P. Vazakidis D. (2020). “Deep learning techniques for stock market prediction in the European union: a systematic review,” in *2020 International Conference on Computational Science and Computational Intelligence (CSCI)*, 605–610.

[ref17] Kim J. Kim H.-S. Choi S.-Y. (2023). Forecasting the S&p 500 index using mathematical-based sentiment analysis and deep learning models: a FinBERT transformer model and LSTM. Axioms 12:835. doi: 10.3390/axioms12090835

[ref18] Kim W. Spörer J. Lee C. L. Handschuh S. (2024). “Is small really beautiful for central Bank communication? Evaluating language models for finance: Llama-3-70B, GPT-4, FinBERT-FOMC, FinBERT, and VADER,” in *5th ACM International Conference on AI in Finance*.

[ref19] Kuiken J. Schuth A. Spitters M. Marx M. (2017). Effective headlines of newspaper articles in a digital environment. Dig. J. 5, 1300–1314. doi: 10.1080/21670811.2017.1279978

[ref20] Kumar G. Jain S. Singh U. P. (2021). Stock market forecasting using computational intelligence: a survey. Arch. Comput. Methods Eng. 28, 1069–1101. doi: 10.1007/s11831-020-09413-5

[ref21] Lai V. Cai J. Z. Tan C. (2019). “Many faces of feature importance: comparing built-in and post-hoc feature importance in text classification,” in *Proceedings of the 2019 Conference on Empirical Methods in Natural Language Processing and the 9th International Joint Conference on Natural Language Processing (EMNLP-IJCNLP)*, 486–495.

[ref22] Latif S. Aslam F. Ferreira P. Iqbal S. (2025). Integrating macroeconomic and technical indicators into forecasting the stock market: a data-driven approach. Economies 13:6. doi: 10.3390/economies13010006, PMID: 40607831

[ref23] Li Y. Jiang S. Li X. Wang S. (2021). The role of news sentiment in oil futures returns and volatility forecasting: data-decomposition based deep learning approach. Energy Econ. 95:105140. doi: 10.1016/j.eneco.2021.105140

[ref24] Li X. Xie H. Chen L. Wang J. Deng X. (2014). News impact on stock price return via sentiment analysis. Knowl.-Based Syst. 69, 14–23. doi: 10.1016/j.knosys.2014.04.022

[ref25] Liagkouras K. Metaxiotis K. (2024). “Extracting sentiment from business news announcements for more efficient decision making” in Advances in artificial intelligence-empowered decision support systems: Papers in honour of professor John Psarras. eds. Tsihrintzis G. A. Virvou M. Doukas H. Lakhmi C. J. (Cham: Springer), 263–282.

[ref26] Liu Z. Huang D. Huang K. Li Z. Zhao J. (2021). “FinBERT: a pre-trained financial language representation model for financial text mining,” in *Proceedings of the Twenty-Ninth International Joint Conference on Artificial Intelligence (IJCAI-20)*, 4513–19.

[ref27] Medhat W. Hassan A. Korashy H. (2014). Sentiment analysis algorithms and applications: a survey. Ain Shams Eng. J. 5, 1093–1113. doi: 10.1016/j.asej.2014.04.011

[ref28] Mishev K. Gjorgjevikj A. Vodenksa I. Chitkushev L. T. Trajanov D. (2020). Evaluation of sentiment analysis in finance: from lexicons to transformers. IEEE Access, 131662–131682. doi: 10.1109/ACCESS.2020.3009626

[ref29] Moran M. (2020). Performance and volatility for sectors in the 2010s. Available online at; https://www.spglobal.com/en/research-insights/articles/performance-and-volatility-for-sectors-in-the-2010s (accessed June 27, 2024).

[ref30] Nti I. K. Adekoya A. F. Weyori B. A. (2020). A systematic review of fundamental and technical analysis of stock market predictions. Artif. Intell. Rev. 53, 3007–3057. doi: 10.1007/s10462-019-09754-z

[ref31] Nuta A. C. Habib A. M. Neslihanoglu S. Dalwai T. Rangu C. M. (2024). Analyzing the market performance of Romanian firms: do the COVID-19 crisis and classification type matter? Int. J. Emerg. Mark. 20:8042. doi: 10.1108/IJOEM-05-2023-0842

[ref32] Rahali A. Akhloufi M. A. (2023). End-to-end transformer-based models in textual-based NLP. AI 4, 54–110. doi: 10.3390/ai4010004

[ref33] Ranco G. Bordino I. Bormetti G. Caldarelli G. Lillo F. Treccani M. (2016). Coupling news sentiment with web browsing data improves prediction of intra-day Price dynamics. PLoS One 11:e0146576. doi: 10.1371/journal.pone.0146576, PMID: 26808833 PMC4726698

[ref34] Reboredo J. C. Ugolini A. (2018). The impact of twitter sentiment on renewable energy stocks. Energy Econ. 76, 153–169. doi: 10.1016/j.eneco.2018.10.014

[ref35] Rieis J. F. de Souza P. Vaz de Melo R. Prates H. K. An J. (2021). Breaking the news: First impressions matter on online news. Proc. Int. AAAI Conf. Web Soc. Media. 9, 357–366. doi: 10.1609/icwsm.v9i1.14619

[ref36] Rouf N. Malik M. B. Arif T. Sharma S. Singh S. Aich S.. (2021). Stock market prediction using machine learning techniques: a decade survey on methodologies, recent developments, and future directions. Electronics 10:2717. doi: 10.3390/electronics10212717, PMID: 40607831

[ref37] Sarkar A. Sahoo A. K. Sah S. Pradhan C. (2020). “LSTMSA: A novel approach for stock market prediction using LSTM and sentiment analysis,” in *2020 International Conference on Computer Science, Engineering and Applications (ICCSEA)* (1–6). IEEE.

[ref38] Shi Y. Tang Y. Cui L. Long W. (2018). A text mining based study of investor sentiment and its influence on stock returns. Econ. Comput. Cybernetics Stu. Res. 52, 183–199. doi: 10.24818/18423264/52.1.18.11

[ref39] US Bank. (2024). Energy sector stocks: is now the time to invest? Available online at: https://www.usbank.com/investing/financial-perspectives/market-news/energy-sector-performance.html (accessed June 27, 2024).

[ref40] Yadav A. Jha C. K. Sharan A. (2020). Optimizing LSTM for time series prediction in Indian stock market. Procedia Comput. Sci. 167, 2091–2100. doi: 10.1016/j.procs.2020.03.257

[ref41] Yoon S. Park K. Lee M. Kim T. Cha M. Jung K. (2021). Learning to detect incongruence in news headline and body text via a graph neural network. IEEE Access 9, 36195–36206. doi: 10.1109/ACCESS.2021.3062029, PMID: 40567712

[ref42] Yoon S. Park K. Shin J. Lim H. Won S. Cha M.. (2019). Detecting incongruity between news headline and body text via a deep hierarchical encoder. AAAI 33, 791–800. doi: 10.1609/aaai.v33i01.3301791

